# Transcriptional Alterations Related to Neuropathology and Clinical Manifestation of Alzheimer’s Disease

**DOI:** 10.1371/journal.pone.0048751

**Published:** 2012-11-07

**Authors:** Aderbal R. T. Silva, Lea T. Grinberg, Jose M. Farfel, Breno S. Diniz, Leandro A. Lima, Paulo J. S. Silva, Renata E. L. Ferretti, Rafael M. Rocha, Wilson Jacob Filho, Dirce M. Carraro, Helena Brentani

**Affiliations:** 1 Research Center (CIPE), A. C. Camargo Hospital, Sao Paulo, Brazil; 2 Memory and Aging Center, Department of Neurology, University of California San Francisco, San Francisco, California, United States of America; 3 Brazilian Brain Bank of the Aging Brain Study Group - Laboratory of Medical Investigations 22 (LIM 22), São Paulo, Brazil; 4 Division of Geriatrics, Medical School, University of Sao Paulo, Sao Paulo, Brazil; 5 Laboratory of Neuroscience - Laboratory of Medical Investigations 27 (LIM 27) - Department and Institute of Psychiatry, Medical School, University of Sao Paulo, Sao Paulo, Brazil; 6 Department of Computer Science, Institute of Mathematics and Statistics, University of Sao Paulo, Sao Paulo, Brazil; 7 Laboratory of Clinical Pathology - Laboratory of Medical Investigations 23 (LIM 23), Department and Institute of Psychiatry, Medical School, University of Sao Paulo, Sao Paulo, Brazil; Nathan Kline Institute and New York University School of Medicine, United States of America

## Abstract

Alzheimer’s disease (AD) is the most common cause of dementia in the human population, characterized by a spectrum of neuropathological abnormalities that results in memory impairment and loss of other cognitive processes as well as the presence of non-cognitive symptoms. Transcriptomic analyses provide an important approach to elucidating the pathogenesis of complex diseases like AD, helping to figure out both pre-clinical markers to identify susceptible patients and the early pathogenic mechanisms to serve as therapeutic targets. This study provides the gene expression profile of postmortem brain tissue from subjects with clinic-pathological AD (Braak IV, V, or V and CERAD B or C; and CDR ≥1), preclinical AD (Braak IV, V, or VI and CERAD B or C; and CDR = 0), and healthy older individuals (Braak ≤ II and CERAD 0 or A; and CDR = 0) in order to establish genes related to both AD neuropathology and clinical emergence of dementia. Based on differential gene expression, hierarchical clustering and network analysis, genes involved in energy metabolism, oxidative stress, DNA damage/repair, senescence, and transcriptional regulation were implicated with the neuropathology of AD; a transcriptional profile related to clinical manifestation of AD could not be detected with reliability using differential gene expression analysis, although genes involved in synaptic plasticity, and cell cycle seems to have a role revealed by gene classifier. In conclusion, the present data suggest gene expression profile changes secondary to the development of AD-related pathology and some genes that appear to be related to the clinical manifestation of dementia in subjects with significant AD pathology, making necessary further investigations to better understand these transcriptional findings on the pathogenesis and clinical emergence of AD.

## Introduction

Alzheimer’s disease (AD) is the most frequent dementing disorder in the elderly and is characterized by a progressive decline in memory and other cognitive domains [1]. The hallmark neuropathological lesions in AD are the presence of neuritic plaques and neurofibrillary tangles, which are secondary to the deposition of β-amyloid peptide (Aβ) and hyperphosphorylated tau protein [2], [3]. The presence of these two lesions is necessary to definite diagnosis of AD in post-mortem studies [4], [5]. The pathogenic mechanisms that lead to AD are unclear. The most accepted pathogenic process in AD derives from the “amyloid cascades hypothesis”. In summary, it states that accumulation of amyloid-β species in the brain, either by increased production or by reduced protein clearance, leads to several secondary pathological changes in neurons and glial cells culminating in widespread neurodegeneration (dystrophic neurites and intracellular neurofibrillary tangles) and cell death [6], [7].

However, there is a growing body of evidence that suggest the accumulation of Aβ in the brain in cognitively healthy older subjects. Post-mortem studies showed that a significant proportion of subjects with neuropathological diagnosis of AD did not have any evidence of cognitive impairment in the last assessment prior to death [8], [9]. More recently, molecular neuroimaging and CSF biomarkers studies demonstrated that 20–40% of older subjects with no cognitive impairment had a significant accumulation of Aβ in the brain [10]–[14]. The most common explanations to such findings are that (1) there is a long pre-clinical phase of AD in which there is the development of brain amyloidosis without apparent clinical symptoms; (2) there are unknown mechanisms of neuronal resilience (brain reserve) or cognitive reserve that protects against the neurotoxic insults related to the amyloidogenesis; (3) the amyloidogenesis observed in AD is part of normal brain aging and some patients may develop or not the dementia syndrome of AD as a consequence of other related neurodegenerative processes. Of note, labeling these individuals as having pre-symptomatic AD is a hypothesis, because some of these individuals will die without ever expressing clinical symptoms. The hypothetical assumption is that asymptomatic individuals with AD pathological changes would have become symptomatic if they lived long enough.

The study of brain mRNA expression profile may help to disentangle some of the molecular mechanisms related to the development of AD-related pathology and the manifestation of clinical dementia in these subjects. Previous studies showed a significant deregulation in the expression of genes related to the energy metabolism [15], transcriptional and tumor suppressor responses [16], apoptosis, inflammation [17], cholinergic activity [18], calcium signaling [19], lipid metabolism [20], and synaptic dysfunction and neuroplasticity [21], [22]. Changes in many of these biological cascades have been demonstrated in peripheral or brain samples of patients with AD or at increased risk to develop dementia [23]–[31]. In addition, these changes were independent of disease severity, according to clinical rating scales. Studies using gene network in this area have contributed to better explore data produced by those mRNA expression studies [32], [33].

Nevertheless, no study to date have addressed whether specific patterns of gene expression is associated to the development of AD-related pathology and to the clinical manifestation of dementia in subjects with significant AD pathology. Therefore, the aims of this study were: (1) to determine which genes and their biological functions are related to the development of AD pathology; (2) to determine which genes and their biological functions are related to the clinical manifestation of dementia in subjects with significant AD pathology.

## Materials and Methods

### Sample Characteristics

After death, a trained gerontologist interviewed a knowledgeable informant who had at least weekly contact with deceased subjects. Past medical history, cognitive performance and functional status was determined for each subject [34]. Dementia stage was ascertained by the Clinical Dementia Rating Scale (CDR) [35]. All informants voluntarily signed an Informed Consent Form and consented to provide all clinical information requested.

At autopsy, hippocampus was dissected and frozen at −80°C. Hippocampal specimens were obtained from the Brain Bank of the Brazilian Aging Brain Study Group [36]. We chose to study only hippocampal gene expression as this brain region shows the earliest pathological changes and hippocampal-mediated episodic memory impairment is the earliest cognitive changes observed in AD patients [37], [38].

Neuropathological examinations were performed using immunohistochemistry according to internationally accepted criteria [36]. Neurofibrillary tangles (NFTs) and neuritic plaques (NPs) were assessed by a skilled neuropathologist in accordance with the Braak and Braak stage system [37], and the Consortium to Establish a Registry for Alzheimer’s Disease (CERAD) [5], respectively. Cases with a Braak stage ≥ IV, or the presence of moderate or frequent neuritic plaques in one or more neocortical regions (CERAD  =  B or C), were classified as meeting criteria for AD. The neuropathologist was blinded to all clinical information.

Based on pathological and clinical criteria, subjects were categorized into three groups: I) 9 subjects with neuropathological AD (Braak ≥ IV and CERAD  =  B or C), and clinical dementia (CDR ≥1), termed “*clinic-pathological* AD” (CP*-*AD); II) 4 subjects with neuropathological AD (Braak ≥IV and CERAD  =  B or C), and without cognitive impairment (CDR = 0), termed “*pathological/preclinical* AD” (P-AD); and III) 10 subjects without neuropathological AD (Braak ≤ II and CERAD 0 or A), and normal cognitive function (CDR = 0), termed “normal older individuals” (N). The neuropathological and clinical data, and post-mortem interval of each case can be visualized in Table 1.

**Table 1:** Summary of selected cases.

Sample ID	Gender	Age	Braak	CERAD	CDR	PMI
CP-AD1	F	99	V	B	3	18.3
CP-AD2	F	82	IV	B	2	13.5
CP-AD3	F	86	IV	C	1	10.1
CP-AD4	F	83	V	A	3	12.1
CP-AD5	M	69	VI	C	2	15.0
CP-AD6	F	87	V	B	3	17.7
CP-AD7	F	82	V	C	2	11.8
CP-AD8	F	77	IV	A	3	16.0
CP-AD9	F	83	VI	C	3	10.8
P-AD1	F	87	V	C	0	11.1
P-AD2	F	85	V	C	0	9.6
P-AD3	M	72	VI	C	0	11.7
P-AD4	F	86	VI	C	0	16.0
N1	F	71	0	0	0	16.1
N2	M	79	I	0	0	8.3
N3	F	81	I	0	0	11.9
N4	M	77	I	A	0	6.5
N5	M	57	0	0	0	9.8
N6	F	65	0	0	0	12.7
N7	F	59	0	0	0	14.0
N8	M	89	II	0	0	14.2
N9	F	82	0	0	0	14.8
N10	F	94	II	0	0	12.3

Subjects were divided in three groups according to neuropathological and clinical criteria: clinic-pathological Alzheimer’s disease (CP-AD), pathological/preclinical Alzheimer’s disease (P-AD), and normal older individuals (N). Sample ID, sample identification; Age, age at death in years; F, female; M, male; Braak, Braak stage; CERAD, Consortium to Establish a Registry for Alzheimeŕs Disease score; CDR, Clinical Dementia Ratio score; PMI, post-mortem interval in hours.

This study was approved by the Ethical Board for Research Project Analysis (CAPPesq) of the University of Sao Paulo Medical School (research protocol 285/04) and was conducted in accordance to the Helsinki Declaration.

### RNA Isolation and Amplification

Total RNA was isolated from the frozen hippocampus by the RNeasy Mini kit (Qiagen, Hilden, Germany) according to the manufacturer’s instructions. RNA purity and yield were determined by UV spectrophotometry for all RNA samples (Table S1). Quality control was performed using the RNA 6000 Pico LabChip® kit with a Model 2100 Bioanalyzer (Agilent Technologies, Waldbronn, Germany). A two-round linear amplification procedure, based on T7-driven amplification, was performed following a previously described protocol [39] with some modifications described below. The total RNA was first denatured at 70°C for 10 minutes in presence of 200 ng oligo dT (24)-T7 primer (5′-AAA CGA CGG CCA GTG AAT TGT AAT ACG ACT CAC TAT AGG CGC T (24)-3′; 57 base pairs) and snap cooled on ice.

Reverse transcription was performed by adding 1× first strand buffer and 0.01 mol/l dithiothrectol (Invitrogen Life Technology, Carlsbad, CA, USA), 2 µl diethylpyrocarbonate (DEPC; Sigma, St Louis, MO, USA) treated water, 40 U rRNasin (Promega, Madison, WI, USA), 1 mmol/l dNTP (Amersham Biosciences, Piscataway, NJ, USA), and 400 U SuperScriptTM II Reverse Transcriptase (Invitrogen Life Technology) to a final volume of 20 µl. The reaction was incubated for 120 minutes at 42°C. Second-strand synthesis was performed by adding 53 µl of DEPC-treated water, 20 µl of 5× second strand buffer (Invitrogen Life Technology), 1 mmol/l dNTP, 1 U RNase H (Invitrogen Life Technology), 10 U *Escherichia coli* DNA ligase, and 40 U *E. coli* DNA polymerase I (Invitrogen Life Technology) to a final volume of 100 µl. The reaction was incubated for 2 hours at 16°C. Ten units of T4 DNA polymerase I (Invitrogen Life Technology) were added and incubated again at 16°C for 5 minutes.

The double strand cDNA (dscDNA) was stopped by adding 0.05 mol/l EDTA. UltraPureTM Phenol (Invitrogen, Carlsbad, CA, USA):chloroform:isoamyl alcohol (Merck), at a ratio of 25∶24∶1 and a pH of 8.0, was used for cDNA purification. The dscDNA was precipitated with absolute ETOH (Merck) and resuspended in 10 µl DEPC-treated water. The dscDNAs were subjected to in vitro transcription using reagents from RibomaxTM Large scale RNA production system T7 kit (Promega), in accordance with the manufacturer’s recommendation. The amplified RNA (aRNA) was reverse transcribed into cDNA using 9 µg random hexamer (dN6; Amersham Bio- sciences, Little Chalfont, UK). cDNA synthesis was continued with the same conditions used in the first strand of the first round. The second strand was synthesized using Advantage® cDNA Polymerase (Clontech, Mountain View, CA, USA), and purification was performed in accordance with the methodology cited above.

The aRNA quality, in terms of purity and integrity (Table S1), was assessed by absorbance at 260/280 nm using a GeneQuant pro spectrophotometer (Amersham Pharmacia Biotech, Little Chalfont, UK) and by electrophoresis in 1% UltraPureTM Agarose (Invitrogen Life Technology) gel, respectively. Only aRNA samples yielding a minimum of 15 µg and presenting a smear concentration between 300 and 700 base pairs (which guarantees high quality hybridization) were further processed. A total RNA pool of 15 cell lines [40] was amplified following the same protocol and used as reference sample for microarray hybridizations.

### cDNA Microarrays and Probes

Labeled cDNA was generated in a reverse transcriptase reaction in the presence of 7 µg of amplified RNA (aRNA), 9 µg of a random hexamer primer (Invitrogen Life Technologies, Carlsbad, CA), Cy3- or Cy5-labeled dCTP (Amersham, Biosciences, Little Chalfont, UK), and 400 U SuperScriptTM II Reverse Transcriptase (Invitrogen Life Technology). The residual dye was removed using illustra AutoSeq™ G-50 (GE Healthcare, Little Chalfont, UK*)*. Equal amounts of test and reference cDNA reverse colored Cy-labeled product were competitively hybridized against the cDNA probes in a customized cDNA platform with 4,608 ORESTES representing human genes [41]. Dye-swap was performed for every sample and used as a replicate. Therefore, 46 arrays were utilized in this study –18 arrays for CP-AD group (9 subjects), 8 arrays for P-AD group (4 subjects), and 20 arrays for N group (10 subjects). Pre-hybridization was carried out in a humidified chamber at 42°C for 6 hours and hybridizations were performed on a GeneTac Hybridization Station (Genome Solutions, MI) at 42°C for 16 to 20 hours.

### Signal Intensity Capture and Analysis

After hybridization, slides were washed as follows: 2× Saline Sodium Citrate (SSC) for 10 minutes, 0.1× SSC/0.1% SDS for 10 minutes (two times), and 0.1× SSC for 10 minutes (two times) at 37°C. All solutions were pre-heated to 42°C. Hybridized arrays were scanned on the ScanArrayTM Express (Packard BioScience Biochip Technologies, Billerica, MA, USA), and Cy5/Cy3 signals were quantified using the histogram method with ScanArray Express software (Perkin-Elmer Life Sciences, Boston, MA, USA). Fluorescent intensities of Cy5 and Cy3 channels on each slide were subjected to spot filtering and normalization. We first eliminated all saturated points (≥63,000; approximately 16 bits) and performed a local background subtraction, considering for analysis only those spots with positives values. Normalization was performed using locally weighted linear regression within and across arrays for inter-slide normalization. After normalization, data for each gene were reported as the logarithm of the expression ratio used to represent the relative gene expression levels in the experimental samples. The raw data from hybridizations and experimental conditions can be obtained at the Gene Expression Omnibus under accession number GSE13214. A detailed description of the platform array is available in accession number GPL1930.

### Statistical Analysis

For analysis of genes related to pathological changes, individuals with AD pathology (CP-AD and P-AD) were compared to individuals without pathology (N). To identify genes implicated with clinical manifestation of dementia, individuals who present AD neuropathology but differ on the clinical status were compared (CP-AD versus P-AD). For both analyses, we first used Student’s *t*-tests with a statistical significance level α = 5% and a False Discovery Rate (FDR) <0.05 to search for differentially expressed genes. As we did not achieve significant results using this criteria, we worked with an α = 1% (without FDR). So, Student’s *t*-tests were carried out at *P*≤0.01 with 1000 permutations by MEV (MultiExperiment Viewer – Boston, MA, USA) software [42]. Genes were functionally classified according to biological processes through Gene Ontology (GO), using FunNet [43]. EntrezGene numbers were used as a standard transcript accession system. All genes of our microarray slide were used as reference set to perform the overrepresentation analysis of the biological GO categories. Significance of overrepresented biological processes was assessed using a built-in Fisher’s exact test with a *P*≤0.05 cut-off. Hierarchical clustering analysis was based on Euclidean distance and average linkage. Reliability of the clustering was assessed by the Bootstrap technique using MEV software.

### Interaction Networks

To see more properties implicated with the differentially expressed genes and their partners, we used a network approach. By querying three human interactome databases (HPRD [44], MINT [45] and IntAct [46]), a protein-protein interaction network starting with those differentially expressed genes was searched, where such genes were mapped in the databases with their first neighbors. Then, we selected only the genes presented on our array platform. To assess differences in network organization between individuals with AD pathology versus normal subjects, we used a nonparametric test to determine the difference in correlation of co-expression of genes with their interactors based on a method analogous to that previously described [47]. First, the Pearson Correlation Coefficient (PCC) of each gene and its interactors for each patient group was calculated. Then the absolute value of the difference of these PCCs was calculated. The magnitude is the difference in PCC of a gene between patient groups. To identify genes that are significantly different between patient groups, we randomly assigned patients to one of two groups and repeated the analysis. This was done 1,000 times to calculate the random distribution. Real PCC differences for genes between patient groups were compared to the random distribution to generate *P*-values (Table S2). This defines a network signature of genes whose co-expression is different as a function of presence or absence of AD pathology. *P*-value cutoff ≤0.05 was considered for significance. The network was visualized using Cytoscape [48].

### Classifiers

Classifiers to separate the CP*-*AD and P*-*AD subjects were searched. To avoid that the classifier could easily be plagued by overfitting due the small number of individuals, we restricted to simple classifiers, in this case linear, with only 3 genes. We also searched for classifiers that present a small bolstered error estimate [49], [50], which was specially developed to deal with very small sample sizes, and usually presents lower variance and bias than traditional techniques, such as leave-one-out or cross-validation [50]. To decrease the computational effort to analyze every possible triplet, we used the pre-processing technique based on linear Support Vector Machines modified to perform feature selection [51]. After pre-processing, around 200 genes were selected. In these genes, a full search was undertaken for triplets, which present a good classification potential based on the bolstered error estimate and distance to the classification surface.

## Results

### Gene Expression Profile Related to AD Pathology

To address which genes were related to the neuropathological processes of AD (“neuropathological AD-related genes”, npADGs), we compared the gene expression profile (Student’s *t*-test, *P*≤0.01) of subjects who have been histopathologically confirmed to demonstrate pathologies associated with AD (Braak stage ≥ IV and CERAD =  B or C, n = 13; representing the CP-AD and P-AD groups) and subjects without evidence of AD-related pathology (Braak stage ≤ II and CERAD = 0 or A, n = 10; representing the N group). A total of 77 genes were differentially expressed - 51 were up-regulated and 26 were down-regulated in AD pathology individuals compared to normal individuals. Differentially expressed genes (DEGs) with their fold change and *P*-values are listed in Table S3. In a subsequent step, we performed a functional analysis that identified biological process categories overrepresented by npADGs (Fisher’s exact test, *P*≤0.05). The Gene Ontology biological process categories are shown in Table 2.

**Table 2:** Biological process categories overrepresented by the genes related to AD neuropathology (npADGs).

regulation of transcription, DNA-dependent (P-value 0.0317)	C-terminal protein amino acid modification (P-value 0.007)
zinc finger protein 266 (*ZNF266*)	isoprenylcysteine carboxyl methyltransferase (*ICMT*)
general transcription factor IIH, polypeptide 1, 62 kDa (*GTF2H1*)	plasminogen activator, urokinase receptor (*PLAUR*)
AF4/FMR2 family, member 3 (*AFF3*)	**RNA export from nucleus (P-value 0.0185)**
zinc finger and BTB domain containing 7B (*ZBTB7B*)	GLE1 RNA export mediator homolog (yeast) (*GLE1*)
helicase-like transcription factor (*HLTF*)	RAE1 RNA export 1 homolog (S. pombe) (*RAE1*)
zinc finger protein 84 (*ZNF84*)	**intermediate filament cytoskeleton organization (P-value 0.0165)**
zinc finger protein 576 (*ZNF576*)	synemin, intermediate filament protein (*SYNM*)
transformation/transcription domain-associated protein (*TRRAP*)	**establishment of localization in cell (P-value 0.0487)**
zinc finger protein 394 (*ZNF394*)	glutamate receptor, ionotropic, kainate 5 (*GRIK5*)
zinc finger protein 559 (*ZNF559*)	**regulation of telomere maintenance (P-value 0.0487)**
lysine (K)-specific demethylase 2B (*KDM2B*)	v-myc myelocytomatosis viral oncogene homolog (avian) (*MYC*)
**chromatin modification (P-value 0.0308)**	**hexose biosynthetic process (P-value 0.0327)**
helicase-like transcription factor (*HLTF*)	carbohydrate (N-acetylgalactosamine 4-sulfate 6-O) sulfotransferase 15 (*CHST15*)
ubiquitin-conjugating enzyme E2A (*UBE2A*)	**DNA synthesis involved in DNA repair (P-value 0.0327)**
transformation/transcription domain-associated protein (*TRRAP*)	Werner helicase interacting protein 1 (*WRNIP1*)
lysine (K)-specific demethylase 2B (*KDM2B*)	**negative regulation of reactive oxygen species metabolic process (P-value 0.0487)**
**mRNA transport (P-value 0.0075)**	acid phosphatase 5, tartrate resistant (*ACP5*)
nucleoporin 50 kDa (*NUP50*)	**acetyl-CoA catabolic process (P-value 0.0327)**
GLE1 RNA export mediator homolog (yeast) (*GLE1*)	nicotinamide nucleotide transhydrogenase (*NNT*)
RAE1 RNA export 1 homolog (S. pombe) (*RAE1*)	**regulation of synaptic vesicle exocytosis (P-value 0.0165)**
**hexose transport (P-value 0.0158)**	glutamate receptor, ionotropic, kainate 5 (*GRIK5*)
nucleoporin 50 kDa (*NUP50*)	**negative regulation of interleukin-1 production (P-value 0.0327)**
RAE1 RNA export 1 homolog (S. pombe) (*RAE1*)	acid phosphatase 5, tartrate resistant (*ACP5*)
**protein targeting to membrane (P-value 0.0089)**	
isoprenylcysteine carboxyl methyltransferase (*ICMT*)	
translocase of inner mitochondrial membrane 9 homolog (yeast) (*TIMM9*)	

Biological process categories significantly overrepresented by npADGs (P<0.05, Fisher’s exact test). Other similar significant categories are not included to reduce redundancy.

Transcription factor (TF) processes were among the largest categories of npADGs. In addition, one of the hallmarks of AD, reduced energy metabolism, was reflected by categories of down-regulated npADGs (*CHST15, NNT, ACP5*) in AD pathology individuals, as hexose biosynthetic process, acetyl-CoA catabolic process and negative regulation of reactive oxygen species metabolic process. Cell adhesion/motility process, comprising *SYMN*, which encodes an intermediate filament responsible for cytoskeleton organization, DNA synthesis/repair process (*WRNIP1*), and telomere maintenance process (*MYC*) were up-regulated in AD pathology individuals. Further, inflammatory processes related to IL-1 and IL-12 production (*ACP5*) were overrepresented. Some DEGs, although not overrepresented in any functional category, must be highlighted such as *ITM2B* that encodes a protein involved in the inhibition and deposition of Aβ [52]; and *PRKCE*, encoder of protein kinase C important to neuron channel activation, which is inhibited by amyloid beta peptide [53].

### Hierarchical Cluster Analysis

Searching for an expression pattern that could differentiate individuals with AD histopathology from normal individuals, a hierarchical clustering was carried out for npADGs (77 genes). However, it was not possible to obtain a clear separation pattern between (CP-AD + P-AD) and N groups (Figure S1).

**Figure 1:**
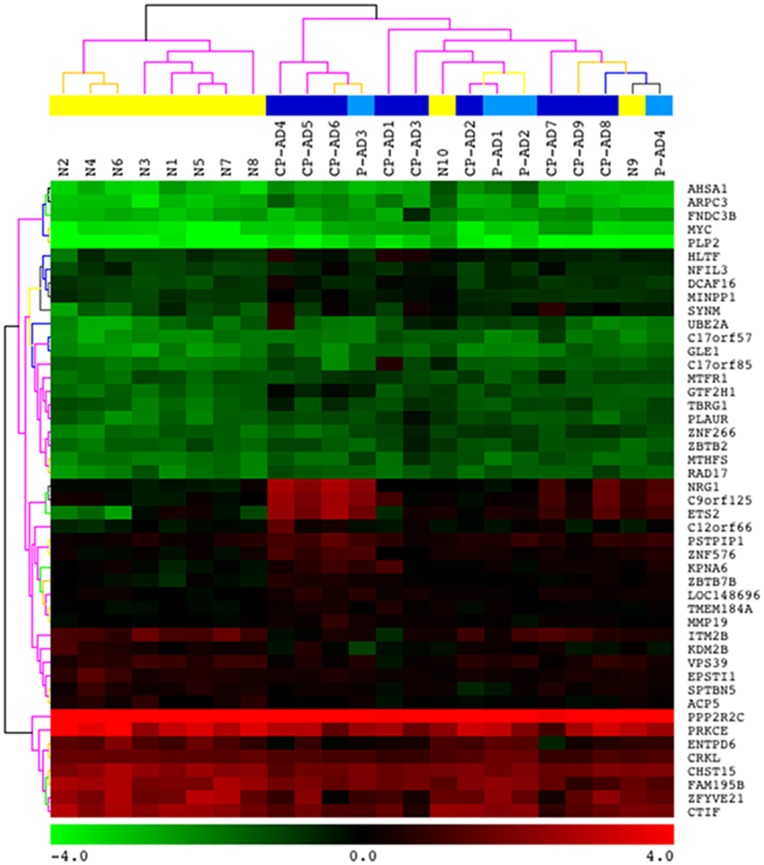
Hierarchical clustering analysis of CP-AD, P-AD and N samples. Hierarchical clustering was performed by using the expression values from the genes related to AD neuropathology with *P*≤0.005 (47 transcripts). Each row represents a single gene and each column a sample (dark blue, CP-AD samples; light blue, P-AD samples; yellow, N samples). Red indicates upregulation, green indicates downregulation, and black indicates no change in expression level comparing to reference sample. Cluster support was given by Bootstrap technic (black, 100% of support; grey, 90–100%; blue, 80–90%; green, 70–80%; light yellow, 60–70%; dark yellow, 50–60%; magenta, 0–50%, red, 0%). CP-AD, clinic-pathological Alzheimer’s disease; P-AD, pathological/preclinical Alzheimer’s disease; N, normal samples (controls).

When the hierarchical clustering was performed using npADGs with *P*≤0.005 (47 genes), clusters of differentially up- and down-regulated genes were identified (Figure 1). The dendrogram shows a discrimination pattern, with 100% of support, in two clusters: 1) AD cluster, which grouped all CP-AD and P-AD samples (13 individuals –100%) plus 2 normal samples (2 out of 10); and 2) N cluster, which grouped just normal samples (8 out of 10). A functional analysis of these 47 genes keeps the overrepresented biological processes identified with all DEGs. Using npADGs with lower *P*-values, the clustering remains the same (data not shown).

### Interaction Network

**Figure 2:**
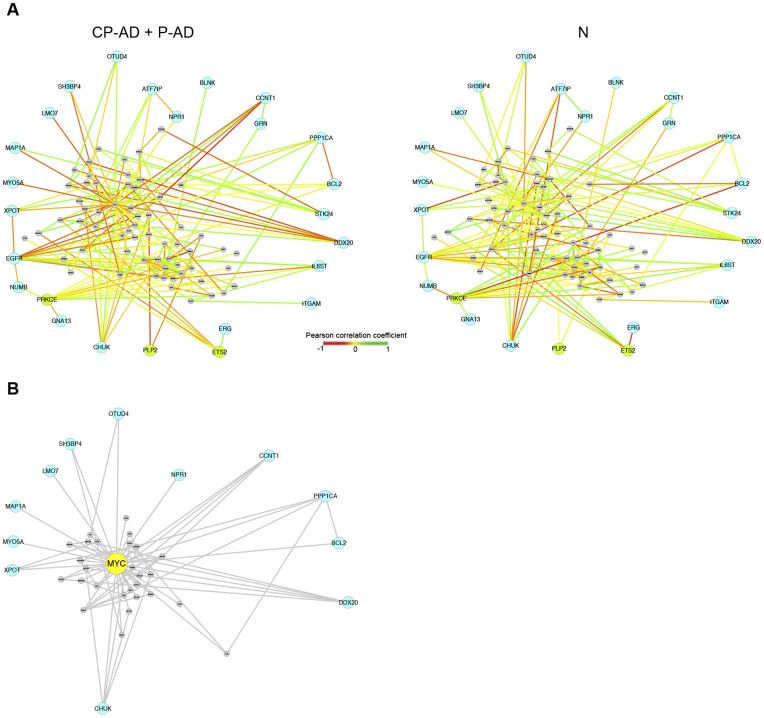
Interaction networks of the significant genes and their interacting partners. (**A**) Shown are the genes (color nodes) that have, as a function of presence (CP-AD + P-AD) or absence (N) of AD pathology, significantly different correlation of co-expression with their partners. Green nodes indicate genes that are significantly differently expressed between patient groups, while light blue nodes indicate genes that are not significantly differently expressed. Edge colors represent the correlation between a gene and each of its partners. (**B**) *MYC* and its interacting partners. Note that the significant genes and their partners form an interconnected network, and despite the interactions involving *MYC* are not significantly altered, it has a lot of connections, playing an important role as a hub gene. CP-AD, clinic-pathological Alzheimer’s disease; P-AD, pathological/preclinical Alzheimer’s disease; N, normal samples (controls).

Aiming to explore the 47 genes identified in the clustering analysis and their connectors an interaction network was constructed. Genes were mapped on the human “interactome” and then only those in our array platform were selected (301 genes). We looked for significant differences in the average PCC of a gene and their interacting partners in subjects who presented AD pathology (CP-AD and P-AD groups) and those who were free of such histopathology. This metric gives an estimate of the difference in correlation of each interaction around a gene between the two groups (AD pathology vs. controls). This revealed 25 genes that displayed altered PCC as a function of presence or absence of AD neuropathology (Figure 2). For instance, one such gene was *PRKCE* that has been shown to be involved with the suppression of Aβ production [54] The expression of *PRKCE* was correlated in a way with the expression of its partners in AD individuals, but changed the correlation with their expression in normal individuals (Figure 2A).

Of the 25 significant genes identified in the network, *PLP2, ETS2,* and *PRKCE* showed significantly differential expression when analyzed using ‘Student’s *t*-test’. On the other hand, some genes with no significant difference in the expression analysis as *BCL2*, gene involved in cell death, had the co-expression of their connectors clearly affected. Besides, genes that have not presented significant difference of co-expression with its partners between the two sample groups can display interesting properties, like *MYC* which plays the role of an important connector gene (Figure 2B), since it has a lot of interacting partners and connects essential parts of the network.

Analysis of interactions between the 25 significant genes and their partners revealed that they form an interconnected network, and a functional analysis of these genes demonstrated overrepresentation of some GO categories involved with transcriptional regulation, DNA damage, inflammatory signaling, cell adhesion, neuron differentiation, and neuron apoptosis (Table S4).

### Gene Expression Profile Related to Clinical Manifestation of AD

When individuals establish substantial neuropathological changes of AD, some of them develop the clinical dementia syndrome, while others remain asymptomatic for a long period, i.e. the preclinical stages of AD. Thus, to assess which genes might be related to the clinical expression of AD (“clinical AD-related genes”, cADGs), we compared the gene expression profile (Student *t*-test, *P*<0.01) of subjects with AD pathology and clinical dementia (CP-AD), and subjects with AD pathology but cognitively normal (P-AD). We found that 23 genes were differentially expressed between these two groups –16 were up-regulated and 7 down-regulated in CP-AD individuals compared to P-AD (Table S5). However, taking into account the small number of DEGs, any gene out of these 23 can be considered differentially expressed by chance due the error of multiple testing. Therefore, a statistically reliable change could not be detected between CP-AD and P-AD groups.

### Classifiers

As our aim was to find out transcriptional differences between CP-AD and P-AD individuals that could not be achieved by differential gene expressions in our study based on our small sample size, we searched for classifiers that were able to completely distinguish these groups utilizing another approach, which did not consider the DEGs, but started using all the genes in the microarray platform and applying a mathematical model design for small sample sizes.

**Figure 3:**
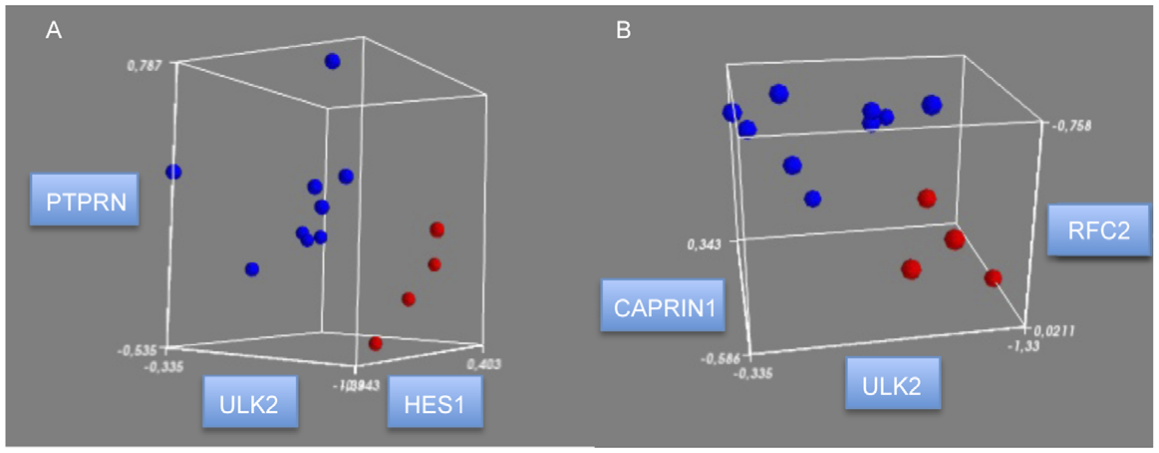
Multivariate (three-gene) discriminators for Alzheimer’s disease (AD) classification. (**A**) Discriminator of CP-AD samples (blue) and P-AD samples (red) using the expression values of *PTPRN*, *ULK2*, and *HES1* genes. (**B**) Discriminator of CP-AD samples (blue) and P-AD samples (red) using the expression values of *CAPRIN1*, *ULK2*, and *RFC2* genes. CP-AD, clinic-pathological Alzheimer’s disease; P-AD, pathological/preclinical Alzheimer’s disease.

Six genes selected by feature selection were used to generate the classifiers between those groups: *CAPRIN1*, *HES1*, *LGR6*, *PTPRN*, *RFC2*, and *ULK2*. A classifier comprised by *PTPRN*, *ULK2*, and *HES1* (Figure 3A) and another classifier comprised by *CAPRIN1*, *ULK2*, and *RFC2* (Figure 3B) were able to classify 100% of samples.

*CAPRIN1* encodes a phosphoprotein required for normal progression through the G1-S phase of the cell cycle [55], and is expressed in post-synaptic granules in neuronal dendrites [56]. Caprin-1 likely regulates transport and translation of mRNAs of proteins involved in synaptic plasticity in neurons [57]. *HES1* is a transcriptional repressor involved in neural development by regulating functional aspects of neural stem cells [58]. *LGR6* encodes a protein that is a glycoprotein hormone receptor. *PTPRN* is a member of the protein tyrosine phosphatase (PTP) family and represents a receptor-type PTP (RPTP). Some ligands for RPTPs play an important role in regulating synaptogenesis and neurite growth [59]. *RFC2* is involved in DNA repair [60]. *ULK2* encodes a protein that is involved in axonal elongation [61].

**Figure 4:**
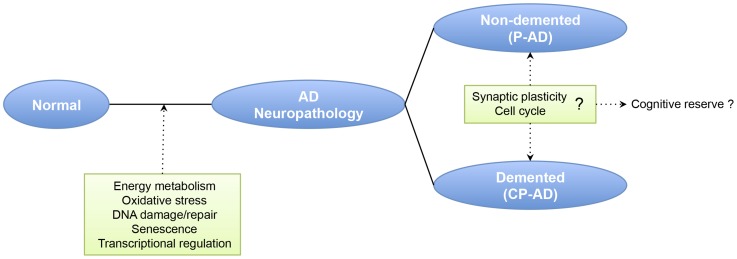
Hypothetical model of the gene expression alterations related to neuropathology and clinical manifestation of Alzheimer’s disease (AD). Gene expression profile changes related to AD pathology are implicated with energy metabolism, oxidative stress, DNA damage and transcriptional regulation. Once established of significant AD pathology, some genes involved with synaptic plasticity, and cell cycle appear to be involved with the clinical outcome of the illness and might represent the molecular mechanisms that underlie the cognitive reserve. CP-AD, clinic-pathological Alzheimer’s disease; P-AD, pathological/preclinical Alzheimer’s disease.

## Discussion

Microarray studies comparing AD subjects with normal elderly individuals have uncovered multiple pathophysiological processes that have been implicated in AD, including energy metabolism [15], transcriptional and tumor suppressor responses [16], apoptosis, inflammation [17], cholinergic activity [18], calcium signaling [19], lipid metabolism [20], and synaptic dysfunction and neuroplasticity [21], [22]. In the present study, we included a group of subjects with preclinical AD - a period during which there are abundant amyloid deposits and neurofibrillary tangles in the brain but no evidence of cognitive decline - to provide further support to the relevance of genes involved in the early development of neurodegenerative changes in AD.

The AD neuropathology-related gene set was involved in some important functions suspected of a role in AD and brain aging, in particular energy metabolism and oxidative stress (hexose transport, hexose biosynthetic process, acetyl-CoA catabolic process, negative regulation of reactive oxygen species metabolic process), immune function (negative regulation of interleukin-1 production), DNA repair (DNA synthesis involved in DNA repair), senescence (regulation of telomere maintenance) and transcriptional regulation (regulation of transcription, DNA-dependent, and chromatin modification). We have to consider that these functions/gene expression might be disrupted as an early response to the increased accumulation of Aβ peptide and tau observed in the CP-AD and P-AD individuals [62].

Glucose metabolism is impaired in AD brain [63], and the decreased neuronal glucose metabolism has been associated with tau hyperphosphorylation [64]. Moreover, histopathological alterations of AD also induce functional deficits of the respiratory chain complexes and therefore consecutively result in mitochondrial dysfunction and oxidative stress [65], [66]. In consequence to the oxidative stress, markers of DNA damage, particularly oxidative DNA damage, have been largely found in brain regions of AD patients. Brain in AD might be subjected to the double insult of increased DNA damage, as well as deficiencies of DNA repair pathways [67]. As regards to telomere maintenance, several studies have addressed the importance of *MYC* in regulating the expression of telomerase [68]. We found *MYC* up-regulated in AD pathology-carrier individuals, which could cause overexpression of telomerase leading to an accentuated ageing cell in such subjects. In relation to immune/inflammatory response, Aβ has proinflammatory actions, including the activation of microglia and stimulating their production and release of inflammatory factors such as IL-1 [69]. Interestingly, the synthesis of amyloid precursor protein (APP), and its cleavage resulting in Aβ peptide, are stimulated by IL-1 [70], [71]. Notably, IL-1 also seems to be implicated with the neurofibrillary tangles, participating at the hyperphosphorylation of tau protein [72]. Of note, Liang et al. [73], comparing neurons of non-demented individuals who demonstrate intermediate levels of AD pathology (Braak stage of II to IV with a CERAD neuritic plaque density of moderate or frequent – similar to P-AD group, but in our case such individuals present high level of pathology) vs. control brains and AD vs. control brains, found common expression changes related to formation of NFTs and amyloid plaques.

From the AD pathology-related DEGs (77 genes), we also identified a gene set (47 genes) providing two clear patterns between individuals with neuropathology and normal subjects. These 47 genes were utilized to construct an interaction network. Network science deals with complexity by “simplifying” complex systems, summarizing them merely as components (nodes) and interactions (edges) between them. The resulting “interactome”, the networks of interactions between cellular components, can serve as scaffold information to extract global or local graph theory properties. Once shown to be statistically different from randomized networks, such properties can then be related back to a better understanding of biological processes [74]. Our interaction network revealed 25 genes and their interactors that showed significant alterations of co-expression of components. Thus, we were able to identify important changes in the network that are associated with the AD neuropathology. An advantage of this approach is the capacity to reveal important genes that were not identified by conventional statistical tests. Furthermore, we could extract some properties of the network, like an important connector gene (hub) represented by *MYC*.

Regarding to genes related to clinical manifestation of dementia in brains with substantial AD histopathology, we compared individuals that have been clinically and histopathologically confirmed to have AD (CP-AD) with individuals who did not fulfill clinical criteria for AD but demonstrate high levels of AD-related pathology (P-AD). With the bias of a limited statistical power, a reliable expression change could not be detected, and so, CP-AD and P-AD were transcriptionally indistinguishable using a statistical test with this small sample size.

However, we utilized a classification approach, which does not consider the differentially expressed genes, but it starts with all genes of the array platform, to discriminate CP-AD and P-AD individuals. Disease classification is another approach already used in the molecular diagnosis and classification of several illnesses, including AD [75], [76]. We found 6 genes capable to separate CP-AD from P-AD individuals. Interestingly, these genes were related to biological functions that have been widely associated with AD, as synaptic plasticity [77]–[80], and cell cycle [81], [82], suggesting them as important pathways on the clinical emergence of the disease. Although we have used a well-established error estimation technique to select good genes and to design classifiers for a small number of samples, we understand that classification approach provides candidates for further validation.

Therefore, we have added some evidence to a hypothesis model in which relatively independent processes contribute to the AD pathology and AD clinical manifestation (Figure 4). The pathological changes are linked to energy metabolism, oxidative stress, DNA damage/repair, senescence and transcriptional regulation. Once developed substantial AD-related pathology, the transcriptional profile between demented and non-demented individuals is very similar, although some genes implicated with synaptic plasticity, and cell cycle might be involved in the clinical manifestation of dementia, requiring further investigations about the roles of these pathways in such subjects. It is relevant to highlight that, since subjects with high burden of histopathological lesions can support them without cognitive decline, the identification of transcriptional alterations in relation to symptomatic AD individuals may help to uncover the molecular basis underlying the cognitive reserve [83]. Another microarray study comparing AD cases and non-demented individuals with AD pathology suggests an immune dysfunction between those groups [84]. Comparing neurons of non-demented individuals with intermediate levels of AD pathology vs. control brains and AD vs. control brains, Liang et al. [73] identified exclusive expression changes related to learning/memory processes in non-demented individuals with AD vs. control comparison, representing possible compensatory efforts targeted against onset of cognitive deficits.

Limitations of this work are comprised by both small sample size and gender unbalance. Searching the DEGs taking out men or women, as in leave-one-out analysis, the final list of genes presents variations independently of gender (data not shown). To overcome these limitations, we used different approaches to find relevant genes: 1) hierarchical clustering and network analysis using DEGs, and 2) classification analysis, not using the DEGs, but starting with all genes in the array.

Further studies with larger sample sizes are necessary to better understand the pathogenic mechanisms of early stages of AD, and to discover pre-clinical biomarkers and rational therapeutic targets. To this end, studies with pre-symptomatic animal models could be of extreme importance on developing of time or stage-dependent interventions to achieve optimal results in delaying the progression of AD-related pathological changes or clinical symptoms of dementia.

## Supporting Information

Figure S1 **Hierarchical clustering was performed by using the expression values from the genes related to AD neuropathology (77 transcripts with** ***P*** **≤0.01).** Each row represents a single gene and each column a sample (dark blue, CP-AD samples; light blue, P-AD samples; yellow, N samples). Red indicates upregulation, green indicates downregulation, and black indicates no change in expression level comparing to reference sample. The cluster support was given by Bootstrap technic (black, 100% of support; grey, 90–100%; blue, 80–90%; green, 70–80%; light yellow, 60–70%; dark yellow, 50–60%; magenta, 0–50%, red, 0%). CP-AD, clinic-pathological Alzheimer’s disease; P-AD, pathological/preclinical Alzheimer’s disease; N, normal samples (controls).
(TIF)

Table S1 **Yield and purity of total and amplified RNA.** RNA purity and yield were determined by UV spectrophotometry. Yield is given in µg and purity was assessed by absorbance at 260/280 nm. CP-AD, clinic-pathological Alzheimer’s disease; P-AD, pathological/preclinical Alzheimer’s disease; N, normal individuals (controls).
(PDF)

Table S2 **Data of interaction network analysis.** To determine the genes and their interactors that significantly discriminate between individuals who demonstrate AD pathology versus those without such lesions we used a non-parametric test. Each gene was assessed for the difference of the Pearson Correlation Coefficient (PCC) of each interaction. Then, the average difference of the absolute value (avg_abs_diff) for the gene and each of interactors was calculated. To determine if the deviation in correlation between the two groups is significant we randomly reassigned the patients to the two groups 1000 times and recalculated the avg_abs_diff. Therefore, the p-value of each gene was given as the frequency of the random avg_abs_diff being greater than the real avg_abs_diff divided by 1000. Original_avg_abs_diff, real average difference of the absolute value of PCCs between (CP-AD + P-AD) vs. N; Degree, number of connectors of a node (gene); Times_greater (in 1000), frequency of the random avg_abs_diff being greater than the real avg_abs_diff.
(PDF)

Table S3 **Identification of the differentially expressed genes related to AD neuropathology (npADGs).** Genes identified by Student’s *t*-test are listed according to *P*-value and the fold change of gene level from the clinic-pathological AD samples + pathological/preclinical AD samples (CP-AD + P-AD) compared to normal samples (N). Genes were considered differentially expressed at *P*-values of ≤0.01. Abbreviations: ORESTES, Open Reading Frame Expressed Sequence Tags identification. GeneBank, accession number at the GeneBank. Entrez Gene, accession number at the Entrez Gene.
(PDF)

Table S4 **Biological process categories overrepresented by the genes related to interaction network.** Biological process categories significantly overrepresented by significant genes and their connectors from the interaction network (*P*≤0.05, Fisher’s exact test). Other similar significant categories are not included to reduce redundancy.
(PDF)

Table S5 **Identification of the differentially expressed genes related to clinical manifestation of AD (cADGs).** Genes identified by Student’s *t*-test are listed according to fold change of gene level and *P*-value from the clinic-pathological AD samples (CP-AD) compared to pathological/preclinical AD samples (P-AD). Genes were considered differentially expressed at *P*≤0.01. Abbreviations: ORESTES, Open Reading Frame Expressed Sequence Tags identification. GeneBank, accession number at the GeneBank. Entrez Gene, accession number at the Entrez Gene.
(PDF)
