# Supplementary figures and images for: Transcriptional Alterations Related to Neuropathology and Clinical Manifestation of Alzheimer’s Disease
Source: PLoS One. 2012 Nov 7;7(11):e48751. doi: 10.1371/journal.pone.0048751 (PMC3492444; doi:10.1371/journal.pone.0048751)

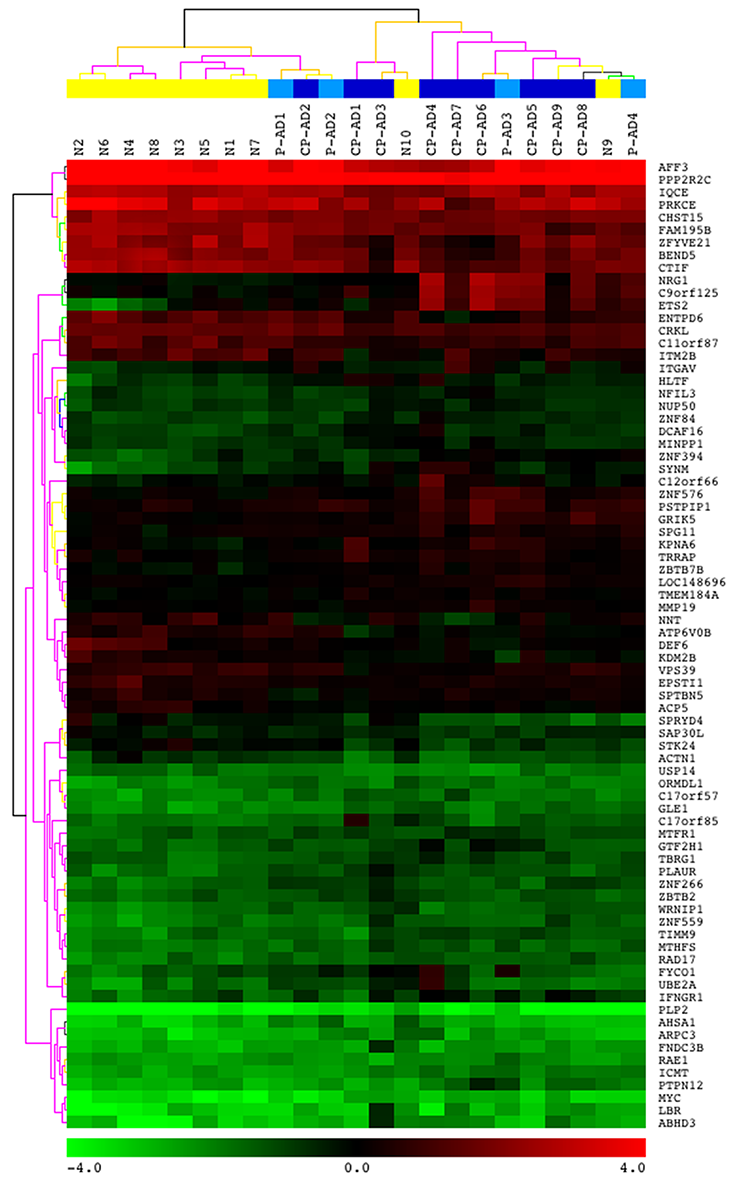

Supplement: Figure S1 — Hierarchical clustering was performed by using the expression values from the genes related to AD neuropathology (77 transcripts with P ≤0.01). Each row represents a single gene and each column a sample (dark blue, CP-AD samples; light blue, P-AD samples; yellow, N samples). Red indicates upregulation, green indicates downregulation, and black indicates no change in expression level comparing to reference sample. The cluster support was given by Bootstrap technic (black, 100% of support; grey, 90–100%; blue, 80–90%; green, 70–80%; light yellow, 60–70%; dark yellow, 50–60%; magenta, 0–50%, red, 0%). CP-AD, clinic-pathological Alzheimer’s disease; P-AD, pathological/preclinical Alzheimer’s disease; N, normal samples (controls). (TIF) [file pone.0048751.s001.tif]
