# Supplementary material for: Transcriptional Alterations Related to Neuropathology and Clinical Manifestation of Alzheimer’s Disease
Source: PLoS One. 2012 Nov 7;7(11):e48751. doi: 10.1371/journal.pone.0048751 (PMC3492444; doi:10.1371/journal.pone.0048751)
Supplement: Table S1 — Yield and purity of total and amplified RNA. RNA purity and yield were determined by UV spectrophotometry. Yield is given in µg and purity was assessed by absorbance at 260/280 nm. CP-AD, clinic-pathological Alzheimer’s disease; P-AD, pathological/preclinical Alzheimer’s disease; N, normal individuals (controls). (PDF) [file pone.0048751.s002.pdf]

Table S1. Yield and purity of total and amplified RNA

	Total RNA		Amplified RNA	
	Yield (µg)	Purity ($R_{260/280}$)	Yield (µg)	Purity ($R_{260/280}$)
CP-AD1	1.77	2.20	38.32	1.95
CP-AD2	1.51	1.92	48.17	2.18
CP-AD3	6.51	1.81	57.88	2.13
CP-AD4	3.06	2.07	94.74	2.01
CP-AD5	2.53	2.03	90.02	2.04
CP-AD6	5.96	2.06	22.88	2.23
CP-AD7	1.31	1.99	37.29	2.27
CP-AD8	8.39	2.07	38.69	2.01
CP-AD9	7.95	1.84	61.38	2.29
P-AD1	3.21	1.82	33.42	2.24
P-AD2	2.01	1.80	47.40	2.20
P-AD3	4.39	2.04	20.10	2.08
P-AD4	1.88	1.80	41.29	2.28
N1	5.28	1.85	42.10	2.22
N2	3.78	2.12	36.21	1.92
N3	4.53	1.81	40.67	2.21
N4	3.57	1.98	27.15	1.88
N5	1.35	1.88	55.68	2.22
N6	7.19	2.05	38.56	1.91
N7	1.38	1.80	28.49	2.18
N8	3.08	2.06	135.41	2.00
N9	3.19	2.02	41.57	2.15
N10	4.62	2.04	51.99	2.33

RNA purity and yield were determined by UV spectrophotometry. Yield is given in µg and purity was assessed by absorbance at 260/280 nm. CP-AD, clinic-pathological Alzheimer's disease; P-AD, pathological/preclinical Alzheimer's disease; N, normal individuals (controls)
