# Supplementary material for: Transcriptional Alterations Related to Neuropathology and Clinical Manifestation of Alzheimer’s Disease
Source: PLoS One. 2012 Nov 7;7(11):e48751. doi: 10.1371/journal.pone.0048751 (PMC3492444; doi:10.1371/journal.pone.0048751)
Supplement: Table S2 — Data of interaction network analysis. To determine the genes and their interactors that significantly discriminate between individuals who demonstrate AD pathology versus those without such lesions we used a non-parametric test. Each gene was assessed for the difference of the Pearson Correlation Coefficient (PCC) of each interaction. Then, the average difference of the absolute value (avg_abs_diff) for the gene and each of interactors was calculated. To determine if the deviation in correlation between the two groups is significant we randomly reassigned the patients to the two groups 1000 times and recalculated the avg_abs_diff. Therefore, the p-value of each gene was given as the frequency of the random avg_abs_diff being greater than the real avg_abs_diff divided by 1000. Original_avg_abs_diff, real average difference of the absolute value of PCCs between (CP-AD + P-AD) vs. N; Degree, number of connectors of a node (gene); Times_greater (in 1000), frequency of the random avg_abs_diff being greater than the real avg_abs_diff. (PDF) [file pone.0048751.s003.pdf]

Table S2. Data of interaction network analysis.

Node (gene)	Original_avg_abs_diff	Degree	Times_greater (in 1000)	P-value
ERG	1.568647	1	1	0.001
PLP2	0.659873	2	3	0.003
ATF7IP	0.702131	4	4	0.004
BLNK	0.763175	1	6	0.006
ETS2	0.936201	6	8	0.008
OTUD4	0.655703	3	13	0.013
BCL2	0.541336	4	16	0.016
LMO7	0.882419	1	16	0.016
DDX20	0.741064	7	21	0.021
MAP1A	0.732543	2	22	0.022
CHUK	0.544891	7	27	0.027
NPR1	0.686389	2	27	0.027
XPOT	0.581697	4	27	0.027
GNA13	0.727478	1	28	0.028
PPP1CA	0.638159	5	33	0.033
NUMB	0.637336	2	34	0.034
GRN	0.552022	2	36	0.036
EGFR	0.514786	19	39	0.039
STK24	0.623512	5	40	0.04
SH3BP4	0.758069	2	42	0.042
MYO5A	0.677371	1	43	0.043
IL6ST	0.422443	5	45	0.045
ITGAM	0.751458	1	46	0.046
CCNT1	0.443448	5	47	0.047
PRKCE	0.525669	12	49	0.049
PXN	0.453182	12	51	0.051
ACTR3	0.828105	1	59	0.059
GTF2H1	0.576209	8	59	0.059
NCOR2	0.571948	5	62	0.062
USP7	0.441348	6	63	0.063
ZMYND11	0.516892	3	68	0.068
TRIM27	0.815606	2	71	0.071
MRPL53	0.778608	1	74	0.074
ATP1A1	0.538344	2	75	0.075
ARPC3	0.82785	2	76	0.076
PGAP1	0.618612	1	78	0.078

Node (gene)	Original_avg_abs_diff	Degree	Times_greater (in 1000)	P-value
STAG2	0.634126	2	85	0.085
USP20	0.602752	2	87	0.087
LTBP4	0.386606	1	90	0.09
GNL3	0.579096	3	94	0.094
EFTUD2	0.543007	4	98	0.098
RB1	0.555705	11	98	0.098
BZW2	0.956946	1	99	0.099
NRXN3	0.665067	1	102	0.102
ITM2B	0.586853	1	104	0.104
MSL1	0.80989	1	106	0.106
FAM190B	0.754508	1	109	0.109
ETNK2	0.639435	1	111	0.111
ARCN1	0.763126	1	113	0.113
ZBTB17	0.596744	2	114	0.114
RNF40	0.472763	2	118	0.118
GTF2E2	0.444312	3	122	0.122
CYBA	0.711609	1	126	0.126
NCOR1	0.461423	7	127	0.127
HAT1	0.62479	1	130	0.13
CRKL	0.467337	23	131	0.131
TUBA1B	0.55746	3	132	0.132
MMP12	0.56598	1	135	0.135
CCR1	0.402568	3	136	0.136
SRP9	0.364854	2	137	0.137
EVL	0.475411	2	143	0.143
MAP7D1	0.430744	2	156	0.156
DNAJA3	0.386569	3	166	0.166
ERC1	0.521555	1	169	0.169
UBE2A	0.509045	7	174	0.174
PSTPIP1	0.475835	8	176	0.176
FTSJ1	0.362276	6	177	0.177
WDR48	0.467662	4	181	0.181
IQCE	0.504713	1	186	0.186
SYNE1	0.53952	1	186	0.186
TEX2	0.583392	1	189	0.189
H2AFZ	0.510105	1	190	0.19
MLL	0.450146	3	196	0.196

Node (gene)	Original_avg_abs_diff	Degree	Times_greater (in 1000)	P-value
PPP2R1B	0.732311	3	204	0.204
DDX1	0.301081	4	205	0.205
ATIC	0.534723	2	206	0.206
TOP2A	0.401574	5	206	0.206
IL1B	0.882566	1	207	0.207
PIK3CB	0.43255	3	208	0.208
BRAF	0.541293	3	209	0.209
ERCC3	0.446474	3	214	0.214
SQRDL	0.387386	2	215	0.215
AKAP8L	0.347017	2	216	0.216
BPTF	0.37061	1	250	0.25
RAD1	0.44458	1	250	0.25
GTF3C4	0.457976	2	252	0.252
AASS	0.38492	1	254	0.254
UBR4	0.487976	2	254	0.254
SLC25A11	0.579928	1	256	0.256
ATM	0.421904	5	257	0.257
BRWD1	0.424583	2	260	0.26
CCT2	0.321198	5	261	0.261
MLH1	0.554082	1	261	0.261
SDF4	0.421357	1	261	0.261
HLTF	0.596814	2	265	0.265
DNM2	0.373333	6	269	0.269
DDX52	0.534252	1	271	0.271
HNRNPA0	0.284347	3	272	0.272
GAB2	0.461858	6	275	0.275
ASXL1	0.634285	1	281	0.281
RBFOX2	0.630827	1	281	0.281
MFHAS1	0.570395	1	283	0.283
KIDINS220	0.499764	3	287	0.287
HSPH1	0.354966	4	289	0.289
KRT1	0.299856	4	289	0.289
KPNB1	0.353463	8	290	0.29
ILVBL	0.475267	1	296	0.296
ARMC6	0.655983	1	297	0.297
AHSA1	0.378707	6	302	0.302
MDN1	0.412825	3	304	0.304

Node (gene)	Original_avg_abs_diff	Degree	Times_greater (in 1000)	P-value
UTP15	0.469484	1	305	0.305
FUBP1	0.415353	2	309	0.309
AIFM2	0.375001	1	311	0.311
CDV3	0.455211	1	316	0.316
GEMIN5	0.383104	3	327	0.327
PRKDC	0.374382	7	332	0.332
UBA1	0.498944	2	336	0.336
RIF1	0.348268	2	337	0.337
PDS5A	0.372726	4	340	0.34
SMAD3	0.370791	13	344	0.344
CREB3	0.328255	1	348	0.348
GNB2L1	0.419455	6	352	0.352
LYAR	0.333917	6	352	0.352
MOGS	0.367848	1	353	0.353
ADIPOR1	0.543291	1	354	0.354
SMARCAD1	0.545519	1	354	0.354
DNAJB6	0.349787	1	357	0.357
CNOT4	0.296979	1	359	0.359
MSH6	0.375883	3	364	0.364
EIF4ENIF1	0.328004	3	370	0.37
TIAM1	0.305519	2	373	0.373
MAP2K1	0.354593	4	376	0.376
FASTKD2	0.381939	1	381	0.381
FERMT3	0.382396	1	381	0.381
CALCOCO2	0.394427	4	383	0.383
LRPPRC	0.33667	4	389	0.389
NPTX2	0.429738	1	389	0.389
TRIM21	0.330153	4	396	0.396
RPRD2	0.452937	1	397	0.397
PLOD3	0.398683	2	398	0.398
E2F1	0.349039	5	399	0.399
RAD18	0.464792	1	400	0.4
KIT	0.350305	5	401	0.401
KNDC1	0.42001	1	402	0.402
XPO7	0.414511	4	402	0.402
GPC4	0.292114	1	416	0.416
SRPX2	0.398687	1	417	0.417

Node (gene)	Original_avg_abs_diff	Degree	Times_greater (in 1000)	P-value
PADI2	0.508418	1	418	0.418
GEMIN4	0.366113	6	420	0.42
PLAU	0.296057	1	424	0.424
CBL	0.401827	12	426	0.426
PPP2R2C	0.469516	4	426	0.426
ETV6	0.380626	3	428	0.428
MYCBP2	0.383138	3	435	0.435
DNAJA2	0.281247	5	445	0.445
ATP6V1H	0.313602	2	454	0.454
ASAP1	0.330033	4	466	0.466
BAHCC1	0.323698	1	476	0.476
LBR	0.235759	2	476	0.476
RANBP2	0.249498	6	477	0.477
SLC25A12	0.326526	2	478	0.478
PLAUR	0.336171	9	482	0.482
DCAF16	0.398039	1	489	0.489
ERBB3	0.345068	5	491	0.491
EXPH5	0.542023	1	491	0.491
PTPN14	0.417623	1	492	0.492
FOSL2	0.334136	1	493	0.493
PGD	0.36641	1	494	0.494
OAT	0.369076	3	499	0.499
LRP1B	0.387327	2	502	0.502
ATP5J2	0.355672	2	503	0.503
ARFGAP1	0.289213	1	520	0.52
IPO7	0.30915	2	529	0.529
SPAG9	0.246619	1	529	0.529
NOL9	0.458427	1	533	0.533
EPHB6	0.373585	2	537	0.537
SMARCA4	0.242868	11	537	0.537
STAT3	0.279557	8	537	0.537
PDLIM4	0.504493	1	541	0.541
GTF3C1	0.305055	2	548	0.548
PPT1	0.27182	1	549	0.549
ACP5	0.240878	2	554	0.554
KIAA2026	0.255116	1	556	0.556
BTAF1	0.232721	1	557	0.557

Node (gene)	Original_avg_abs_diff	Degree	Times_greater (in 1000)	P-value
MSH2	0.292467	4	560	0.56
PTPN12	0.27404	7	564	0.564
FANCI	0.324683	4	572	0.572
IPO13	0.297875	1	573	0.573
BUB1	0.248054	1	574	0.574
EPOR	0.345744	8	574	0.574
MAP1LC3B	0.262384	19	575	0.575
FAM122B	0.289379	2	578	0.578
PFDN5	0.301529	2	580	0.58
EXOSC1	0.259845	1	584	0.584
MYO1D	0.33512	3	597	0.597
MYC	0.326574	200	601	0.601
FAF2	0.20431	3	603	0.603
PTGES2	0.265362	1	603	0.603
PSMC2	0.306101	3	604	0.604
RELB	0.215126	9	609	0.609
ERCC5	0.354129	2	614	0.614
SMTN	0.193116	1	616	0.616
MINA	0.265456	1	619	0.619
CLPB	0.322545	1	620	0.62
SEC11C	0.292646	1	625	0.625
IL16	0.286634	1	628	0.628
TCF12	0.209287	2	629	0.629
POLR2A	0.207805	4	634	0.634
BRF2	0.299822	14	639	0.639
CYR61	0.191726	1	642	0.642
TNFSF10	0.189642	1	642	0.642
IKBKAP	0.268342	5	647	0.647
PPP1R15A	0.323648	4	647	0.647
MAP4K5	0.247155	2	654	0.654
SNRNP70	0.278033	3	654	0.654
PSMD2	0.282269	4	658	0.658
EHHADH	0.228872	1	660	0.66
FZR1	0.195903	1	688	0.688
ITGB1	0.281382	7	692	0.692
NEDD9	0.28766	6	692	0.692
NOP56	0.167863	1	692	0.692

Node (gene)	Original_avg_abs_diff	Degree	Times_greater (in 1000)	P-value
TRRAP	0.206753	5	692	0.692
TRMT1L	0.154615	1	699	0.699
CCT5	0.267057	5	703	0.703
EP400	0.213493	3	711	0.711
UBR3	0.116811	1	711	0.711
DDB1	0.257434	4	712	0.712
ZFP106	0.206892	1	712	0.712
PPP2R1A	0.338627	7	719	0.719
PECAM1	0.262982	3	726	0.726
PLOD2	0.138959	1	728	0.728
PTP4A3	0.257496	21	731	0.731
MAP4K4	0.225708	2	735	0.735
RFC2	0.232907	3	735	0.735
TRAK1	0.121648	2	735	0.735
PTPN11	0.297564	15	758	0.758
GTF3C3	0.207238	2	759	0.759
DDX21	0.234496	5	763	0.763
DOCK7	0.169365	2	763	0.763
SORBS1	0.221122	4	764	0.764
LIMK1	0.179953	2	766	0.766
AP3D1	0.115533	1	769	0.769
DDX24	0.256868	4	774	0.774
SH3RF1	0.132499	1	774	0.774
ARL1	0.19693	2	775	0.775
ADAP1	0.222588	1	780	0.78
LEPRE1	0.267027	3	780	0.78
RBM10	0.16287	1	780	0.78
SCD	0.148436	1	783	0.783
ZC3H7B	0.198789	1	785	0.785
NUP188	0.194946	3	788	0.788
PSMD3	0.236257	4	790	0.79
TMEM33	0.176389	4	812	0.812
LAS1L	0.092995	1	819	0.819
NEK6	0.228225	8	820	0.82
PCBP1	0.113142	1	820	0.82
GAD1	0.127755	1	821	0.821
IMMT	0.217102	3	825	0.825

Node (gene)	Original_avg_abs_diff	Degree	Times_greater (in 1000)	P-value
PTPN18	0.152532	2	829	0.829
UNC45A	0.170866	3	833	0.833
CHST15	0.106232	1	837	0.837
QPCTL	0.086449	1	839	0.839
SF3B1	0.141153	2	839	0.839
MCM7	0.224889	6	843	0.843
ACTL6A	0.152217	7	845	0.845
NUP133	0.097604	1	852	0.852
XPO5	0.166447	3	857	0.857
RPL3	0.199939	4	860	0.86
ELF3	0.216581	4	863	0.863
ZC3H18	0.074061	1	863	0.863
SEC31B	0.065758	1	885	0.885
KANK2	0.066353	1	886	0.886
POLD1	0.172762	3	886	0.886
MAST4	0.053707	1	897	0.897
NUP93	0.1601	5	905	0.905
KPNA6	0.186215	6	907	0.907
PPP6C	0.050561	1	914	0.914
OBSCN	0.110133	2	917	0.917
DNAJB11	0.112256	2	919	0.919
IL4R	0.203155	4	919	0.919
MRPL14	0.051281	1	920	0.92
SHC1	0.242627	18	923	0.923
KALRN	0.044609	1	928	0.928
SBNO2	0.051855	1	928	0.928
SNX19	0.036985	1	929	0.929
SMARCB1	0.156795	8	941	0.941
EP300	0.224514	13	943	0.943
ATP5O	0.031593	1	950	0.95
NEK9	0.147813	5	950	0.95
WDR77	0.092458	3	957	0.957
PPIP5K2	0.033503	1	958	0.958
TAF2	0.083358	2	961	0.961
HCK	0.204957	14	972	0.972
TTN	0.103785	3	984	0.984
RAPGEF1	0.13681	5	985	0.985

Node (gene)	Original_avg_abs_diff	Degree	Times_greater (in 1000)	P-value
ANLN	0.007435	1	987	0.987
GABARAPL2	0.177253	29	989	0.989
GAB1	0.167027	8	992	0.992
ST6GALNAC6	0.002699	1	997	0.997
KAT2A	0.057523	5	1000	1
LRP1	0.009551	2	1000	1

To determine the genes and their interactors that significantly discriminate between individuals who demonstrate AD pathology versus those without such lesions we used a non-parametric test. Each gene was assessed for the difference of the Pearson Correlation Coefficient (PCC) of each interaction. Then, the average difference of the absolute value (avg_abs_diff) for the gene and each of interactors was calculated. To determine if the deviation in correlation between the two groups is significant we randomly reassigned the patients to the two groups 1000 times and recalculated the avg_abs_diff. Therefore, the p-value of each gene was given as the frequency of the random avg_abs_diff being greater than the real avg_abs_diff divided by 1000. Original_avg_abs_diff, real average difference of the absolute value of PCCs between CP-AD + P-AD vs. N; Degree, number of connectors of a node (gene); Times_greater (in 1000), frequency of the random avg_abs_diff being greater than the real avg_abs_diff.
