# Supplementary material for: Transcriptional Alterations Related to Neuropathology and Clinical Manifestation of Alzheimer’s Disease
Source: PLoS One. 2012 Nov 7;7(11):e48751. doi: 10.1371/journal.pone.0048751 (PMC3492444; doi:10.1371/journal.pone.0048751)
Supplement: Table S3 — Identification of the differentially expressed genes related to AD neuropathology (npADGs). Genes identified by Student’s t-test are listed according to P-value and the fold change of gene level from the clinic-pathological AD samples + pathological/preclinical AD samples (CP-AD + P-AD) compared to normal samples (N). Genes were considered differentially expressed at P-values of ≤0.01. Abbreviations: ORESTES, Open Reading Frame Expressed Sequence Tags identification. GeneBank, accession number at the GeneBank. Entrez Gene, accession number at the Entrez Gene. (PDF) [file pone.0048751.s004.pdf]

Table S3. Identification of the differentially expressed genes related to AD neuropathology (npADGs).

ORESTES	GenBank	Gene Symbol	Entrez Gene	Name	*P*-Value	Fold
CM1-BT0019-231299-074-f10	AW369582	GTF2H1	2965	General transcription factor IIH, polypeptide 1, 62kDa	0	1.43
MR3-GN0186-171100-007-d07	BQ367148	TBRG1	84897	Transforming growth factor beta regulator 1	0	1.32
MR3-HN0062-181200-004-a01	BF829157	KDM2B	84678	Lysine (K)-specific demethylase 2B	0	-1.34
PM0-HT0018-170899-001-a03	AW177711	ETS2	2114	V-ets erythroblastosis virus E26 oncogene homolog 2 (avian)	0	2.92
QV0-BN0041-310300-180-f09	AW995236	CHST15	51363	Carbohydrate (N-acetylgalactosamine 4-sulfate 6-O) sulfotransferase 15	0	-1.33
QV1-CI0175-021100-452-h05	BF803592	VPS39	23339	Vacuolar protein sorting 39 homolog (S. cerevisiae)	0	-1.27
QV3-SN0022-010600-218-e02	BE841175	C17orf57	124989	Hypothetical protein LOC124989	0	1.33
QV4-NT0040-170400-174-g03	AW890450	SYNM	23336	Synemin, intermediate filament protein	0	1.93
CM1-TN0141-250900-439-c04	BF377900	C17orf85	55421	Chromosome 17 open reading frame 85	0.001	1.28
CM3-FT0096-140700-243-f08	BE771837	CTIF	9811	CBP80/20-dependent translation initiation factor	0.001	-1.72
MR3-GN0187-201100-010-f03	BG003841	ZBTB7B	51043	Zinc finger and BTB domain containing 7B	0.001	1.26
MR4-BT0358-130700-010-h10	BE693648	MTHFS	10588	5,10-methenyltetrahydrofolate synthetas	0.001	1.29
PM2-MT0044-010700-001-e07	BE831258	ARPC3	10094	Actin related protein 2/3 complex, subunit 3, 21kDa	0.001	1.36
QV0-DT0047-170200-123-g09	AW938004	ACP5	54	Acid phosphatase 5, tartrate resistant	0.001	-1.26
QV2-FT0010-010700-252-g07	BE768509	HLTF	6596	Helicase-like transcription factor	0.001	1.55
RC0-HT0374-131299-031-c12	AW384219	MYC	4609	V-myc myelocytomatosis viral oncogene homolog (avian)	0.001	1.51
RC2-CS0018-250900-011-b04	BF229778	ZBTB2	57621	Zinc finger and BTB domain containing 2	0.001	1.22
RC4-CN0050-140100-011-a11	AW604072	PLAUR	5329	Plasminogen activator, urokinase receptor	0.001	1.35
MR0-CT0451-211100-021-g07	BF758919	ENTPD6	955	Ectonucleoside triphosphate diphosphohydrolase 6 (putative)	0.002	-1.66
MR0-HT0167-141199-002-h03	BE144581	ZFYVE21	79038	Zinc finger, FYVE domain containing 21	0.002	-1.80
MR0-OT0082-230200-001-d09	AW886500	DCAF16	54876	DDB1 and CUL4 associated factor 16	0.002	1.29
MR3-GN0186-171100-007-d08	BG003230	MINPP1	9562	Multiple inositol-polyphosphate phosphatase 1	0.002	1.21
MR4-EN0075-040501-007-a09	BQ329616	SPTBN5	51332	Spectrin, beta, non-erythrocytic 5	0.002	-1.23
MR4-TN0110-080900-201-d01	BQ374975	FNDC3B	64778	fibronectin type III domain containing 3B	0.002	1.28
QV4-MT0067-270600-268-e02	BE831904	FAM195B	348262	Family with sequence similarity 195, member B	0.002	-1.42
QV4-UM0094-160300-136-g07	BF378928	NFIL3	4783	nuclear factor, interleukin 3 regulated	0.002	1.26
RC4-GN0139-131100-021-d04	BQ367012	RAD17	5884	RAD17 homolog isoform 1	0.002	1.31
CM3-CT0341-170200-093-c07	AW858463	EPSTI1	94240	Epithelial stromal interaction 1 (breast)	0.003	-1.23
MR3-GN0187-171100-011-h04	BG003089	TMEM184A	202915	Transmembrane protein 184A	0.003	1.12
PM0-GN0210-101100-001-c12	BF998151	LOC148696	148696	Hypothetical LOC148696	0.003	1.15
RC2-HT0560-190200-011-b02	BE173404	GLE1	2733	GLE1 RNA export mediator homolog (yeast)	0.003	1.29
CM1-NT0242-271200-679-g10	BF935252	PSTPIP1	9051	Proline-serine-threonine phosphatase interacting protein 1	0.004	1.33
MR0-BN0115-150300-002-e11	BE005088	C12orf66	144577	Hypothetical protein LOC144577	0.004	1.32
MR2-NN1112-290700-004-e10	BE703712	PPP2R2C	5522	Protein phosphatase 2, regulatory subunit B, gamma	0.004	-1.50
MR4-TN0114-110900-101-e07	BF092878	ITM2B	9445	Integral membrane protein 2B	0.004	-1.48
PM3-HT0175-300999-001-g08	BQ356322	NRG1	3084	Neuregulin 1 isoform HRG-beta2b	0.004	2.16
QV3-ET0063-090800-291-e01	BE829325	MMP19	4327	Matrix metallopeptidase 19	0.004	1.12
RC0-CN0028-090200-031-f11	AW842249	ZNF266	10781	Zinc finger protein 266	0.004	1.40
RC2-TN0125-150800-012-g04	BE844225	MTFR1	9650	Mitochondrial fission regulator 1 isoform 1	0.004	1.30
RC3-CN0018-020800-016-f03	BE825264	UBE2A	7319	Ubiquitin-conjugating enzyme E2A (RAD6 homolog)	0.004	1.51
IL3-HT0060-200899-009-B06	AW177993	AHSA1	10598	AHA1, activator of heat shock 90kDa protein ATPase homolog 1 (yeast)	0.005	1.24
MR2-NT0136-161100-004-g03	BF920912	CRKL	1399	V-crk sarcoma virus CT10 oncogene homolog (avian)-like	0.005	-1.21
PM0-CT0153-010999-001-c07	AW752098	C9orf125	84302	Chromosome 9 open reading frame 125	0.005	2.07

ORESTES	GenBank	Gene Symbol	Entrez Gene	Name	*P*-Value	Fold
QV0-CT0224-221199-057-f10	AW752983	PLP2	5355	Proteolipid protein 2 (colonic epithelium-enriched)	0.005	1.27
QV3-DT0045-221299-046-f05	AW364861	ZNF576	79177	Zinc finger protein 576	0.005	1.32
QV4-GN0122-131000-467-g06	BF987914	PRKCE	5581	Protein kinase C, epsilon	0.005	-1.72
RC2-HT0221-061199-031-e09	AW378473	KPNA6	23633	Karyopherin alpha 6 (importin alpha 7)	0.005	1.36
CM0-BN0103-160300-291-d02	BE004154	ACTN1	87	Actinin, alpha 1 isoform a	0.006	-1.56
MR2-NT0138-301100-004-h05	BF923962	AFF3	3899	AF4/FMR2 family, member 3	0.006	-1.69
QV2-CI0105-261200-591-c05	BF819045	LBR	3930	Lamin B receptor	0.006	1.59
QV3-GN0061-160800-296-b07	BE930145	STK24	8428	Serine/threonine kinase 24	0.006	-1.56
RC0-HT0841-150800-021-g04	BE719302	BEND5	79656	BEN domain containing 5	0.006	-1.57
CM1-NT0270-271200-678-h01	BF935421	GRIK5	2901	Glutamate receptor, ionotropic, kainate 5	0.007	1.29
MR0-HT0240-081199-001-b06	AW379158	ZNF559	84527	Zinc finger protein 559	0.007	1.36
MR0-HT0407-140100-002-g02	AW606669	FYCO1	79443	FYVE and coiled-coil domain containing 1	0.007	1.52
MR2-HT0757-270600-013-h11	BE716019	ATP6V0B	533	ATPase, H+ transporting, lysosomal 21kDa, V0	0.007	-1.36
QV1-ET0180-021200-524-h01	BF881076	IFNGR1	3459	Interferon gamma receptor 1	0.007	1.58
QV2-BT0258-141299-020-h11	AW370650	SPRYD4	283377	SPRY domain containing 4	0.007	-1.76
QV2-OT0062-080500-203-h01	AW883691	IQCE	23288	IQ motif containing E	0.007	-1.27
CM0-TN0034-100800-493-g10	BF376181	ITGAV	3685	Integrin alpha-V isoform 1 precursor	0.008	1.45
MR0-BT0551-060300-102-h03	BE073224	ICMT	23463	Isoprenylcysteine carboxyl methyltransferase	0.008	1.32
MR0-NT0039-160400-001-b08	AW890224	DEF6	50619	Differentially expressed in FDCP 6 homolog (mouse)	0.008	-1.52
MR2-CN0035-220300-204-e05	AW842619	ABHD3	171586	Abhydrolase domain containing 3	0.008	1.90
PM0-NN0046-040400-001-h11	AW896256	C11orf87	399947	Chromosome 11 open reading frame 87	0.008	-1.32
PM2-HT0130-150999-001-b08	BE141963	ZNF394	84124	Zinc finger protein 394	0.008	1.60
QV0-AN0048-010800-325-h11	BE812623	TRRAP	8295	Transformation/transcription domain-associated protein	0.008	1.23
QV3-ET0171-231100-492-a10	BF879244	USP14	9097	Ubiquitin specific peptidase 14 (tRNA-guanine transglycosylase)	0.008	-1.25
RC2-ET0019-200600-017-f12	BF358810	WRNIP1	56897	Werner helicase interacting protein 1	0.008	1.24
MR0-BN0115-250900-011-b02	BF154492	NUP50	10762	Nucleoporin 50kDa	0.009	1.17
PM2-CI0111-091100-002-g08	BF806819	SPG11	80208	Spastic paraplegia 11 (autosomal recessive)	0.009	1.17
QV1-GN0207-131100-489-b09	BG001122	PTPN12	5782	Protein tyrosine phosphatase, non-receptor type 12	0.009	1.65
RC1-GN0198-011200-013-e09	BG009053	ZNF84	7637	Zinc finger protein 84	0.009	1.31
MR0-HT0559-290500-026-f04	BE708226	NNT	23530	Nicotinamide nucleotide transhydrogenase	0.01	-1.46
MR3-GN0187-271100-016-b05	BQ367273	SAP30L	79685	SAP30-like isoform 1	0.01	-1.24
MR3-ST0290-110100-201-b10	AW582658	TIMM9	26520	Translocase of inner mitochondrial membrane 9 homolog (yeast)	0.01	1.35
QV4-OT0035-020300-124-b03	AW881364	RAE1	8480	RAE1 RNA export 1 homolog (S. pombe)	0.01	1.17
RC2-BT0642-130300-017-f05	BQ302945	ORMDL1	94101	ORM1-like 1	0.01	1.19

Genes identified by Student t-test are listed according to *P*-value and the fold change of gene level from the clinic-pathological AD samples + pathological/preclinical AD samples (CP-AD + P-AD) compared to normal samples (N). Genes were considered differentially expressed at *P*-values of ≤ 0.01. Abbreviations: ORESTES, Open Reading Frame Expressed Sequence Tags identification. GeneBank, accession number at the GeneBank. Entrez Gene, accession number at the Entrez Gene.
