# Supplementary material for: Transcriptional Alterations Related to Neuropathology and Clinical Manifestation of Alzheimer’s Disease
Source: PLoS One. 2012 Nov 7;7(11):e48751. doi: 10.1371/journal.pone.0048751 (PMC3492444; doi:10.1371/journal.pone.0048751)
Supplement: Table S4 — Biological process categories overrepresented by the genes related to interaction network. Biological process categories significantly overrepresented by significant genes and their connectors from the interaction network (P≤0.05, Fisher’s exact test). Other similar significant categories are not included to reduce redundancy. (PDF) [file pone.0048751.s005.pdf]

Table S4. Biological process categories overrepresented by the genes related to interaction network.

Biological processes	Genes	P-Value
signal transduction	GNA13, DNM2, EGFR, ERBB3, ERG, GRN, ATM, PECAM1, PIK3CB, PLAUR, PRKCE, MAP2K1, PXN, STAT3, STK24, IKBKAP	0.00161
apoptosis	EP300, PPP1R15A, KPNB1, SMAD3, MLL, BCL2, TIAM1, STK24, DNAJA3, ITM2B	0.02360
transcription, DNA-dependent	ELF3, ETS2, SMAD3, MLL, MYC, RB1, RELB, STAT3, CBL	0.01040
nerve growth factor receptor signaling pathway	CHUK, PIK3CB, PRKCE, MAP2K1, SHC1, BRAF, STAT3, TIAM1	0.00068
epidermal growth factor receptor signaling pathway	EGFR, GAB1, MAP2K1, PTPN11, PXN, SHC1, CBL	0.00002
cytokine-mediated signaling pathway	CCR1, IL6ST, PLP2, PTPN11, STAT3, DNAJA3	0.00126
cell-cell adhesion	EGFR, ITGB1, PIK3CB, BCL2, SHC1	0.00539
positive regulation of MAP kinase activity	EGFR, PIK3CB, MAP2K1, SHC1	0.01200
neuron apoptosis	ERBB3, ATM, RB1, BCL2	0.00010
response to DNA damage stimulus	PPP1R15A, ATM, BCL2	0.03670
response to oxidative stress	KRT1, MAP2K1, BCL2	0.03670
neuron differentiation	MAP2K1, RB1, SHC1	0.01220

Biological process categories significantly overrepresented by significant genes and their connectors from the interaction network ($P < 0.05$, Fisher's exact test). Other similar significant categories are not included to reduce redundancy.
