# Supplementary material for: Transcriptional Alterations Related to Neuropathology and Clinical Manifestation of Alzheimer’s Disease
Source: PLoS One. 2012 Nov 7;7(11):e48751. doi: 10.1371/journal.pone.0048751 (PMC3492444; doi:10.1371/journal.pone.0048751)
Supplement: Table S5 — Identification of the differentially expressed genes related to clinical manifestation of AD (cADGs). Genes identified by Student’s t-test are listed according to fold change of gene level and P-value from the clinic-pathological AD samples (CP-AD) compared to pathological/preclinical AD samples (P-AD). Genes were considered differentially expressed at P≤0.01. Abbreviations: ORESTES, Open Reading Frame Expressed Sequence Tags identification. GeneBank, accession number at the GeneBank. Entrez Gene, accession number at the Entrez Gene. (PDF) [file pone.0048751.s006.pdf]

Table S5. Identification of the differentially expressed genes related to clinical manifestation of AD (cADGs).

ORESTES	GenBank	Gene Symbol	Entrez Gene	Gene Name	Fold	P-Value
QV1-ET0058-190600-243-f11	BE828861	LPCAT2	54947	Lysophosphatidylcholine acyltransferase 2	1.71	0.01
CM0-BT0794-030600-412-f11	BE695309	C7orf44	55744	Chromosome 7 open reading frame 44	1.58	0.002
QV2-BT0685-090800-298-h02	BE694304	MLL3	58508	Myeloid/lymphoid or mixed-lineage leukemia 3	1.57	0.008
QV4-HT0691-270400-186-c10	BE184798	PPP2R1B	5519	Protein phosphatase 2, regulatory subunit A, beta	1.33	0.01
QV3-SN0022-010600-218-e02	BE841175	C17orf57	124989	Hypothetical protein LOC124989	1.32	0.007
MR3-UT0091-150900-006-g06	BF096179	TMCC1	23023	Transmembrane and coiled-coil domain family 1	1.32	0.01
RC0-BN0280-310700-025-h02	BF329787	GTF2I	2969	General transcription factor IIi isoform 1	1.29	0.007
QV3-HT0514-150300-119-e11	BE168329	CLN5	1203	Ceroid-lipofuscinosis, neuronal 5	1.27	0.008
CM1-HT0878-031000-453-c05	BQ354196	PPP2CB	5516	Protein phosphatase 2, catalytic subunit, beta	1.27	0.01
RC2-BN0332-200600-011-e03	BE819480	PTTG1IP	754	Pituitary tumor-transforming 1 interacting protein	1.25	0.008
QV3-OT0063-270400-165-g07	AW884072	CPZ	8532	Carboxypeptidase Z	1.25	0.007
QV2-NN0045-041000-403-c05	BF943256	DCLK2	166614	Doublecortin-like kinase 2	1.21	0.009
RC1-BT0254-271100-121-g01	BQ304768	SERPINA1	5265	Serpin peptidase inhibitor, clade A (alpha-1 antiproteinase, antitrypsin), member 1	1.20	0.01
MR3-GN0187-201100-010-d10	BG003866	LRRC41	10489	Leucine rich repeat containing 41	1.18	0.002
RC3-CT0413-270700-022-h11	BF335033	MACC1	346389	Metastasis associated in colon cancer 1	1.18	0.005
QV3-BT0381-161299-042-e07	AW372944	PATZ1	23598	POZ (BTB) and AT hook containing zinc finger 1	1.14	0.006
CM0-ET0122-311000-657-d07	BF871188	TBC1D10A	83874	TBC1 domain family, member 10A	-1.19	0.008
QV4-CI0098-131000-462-c01	BF799785	BRIP1	83990	BRCA1 interacting protein C-terminal helicase 1	-1.23	0.008
QV3-HT1017-231100-481-f08	BF837872	MRPL14	64928	Mitochondrial ribosomal protein L14	-1.23	0.006
QV1-CI0173-061100-460-c12	BF805201	CECR5	27440	Cat eye syndrome chromosome region, candidate 5	-1.51	0.002
QV1-HT0516-140300-107-f06	BE168732	BSCL2	26580	Berardinelli-Seip congenital lipodystrophy 2 (seipin)	-1.60	0.009
QV2-OT0062-010500-177-f06	AW883191	ZCCHC17	51538	Zinc finger, CCHC domain containing 17	-1.96	0.001
MR1-HT0710-220500-002-a04	BE713379	KLK7	5650	Kallikrein-related peptidase 7	-4.01	0.006

Genes identified by Student t-test are listed according to fold change of gene level and P-value from the clinic-pathological AD samples (CP-AD) compared to pathological/preclinical AD samples (P-AD). Genes were considered differentially expressed at *P* Values of ≤ 0.01. Abbreviations: ORESTES, Open Reading Frame Expressed Sequence Tags identification. GeneBank, accession number at the GeneBank. Entrez Gene, accession number at the Entrez Gene.
